# Challenges in the Management of the Patient with a Failing Kidney Graft: A Narrative Review

**DOI:** 10.3390/jcm11206108

**Published:** 2022-10-17

**Authors:** Rita Leal, Clara Pardinhas, António Martinho, Helena Oliveira Sá, Arnaldo Figueiredo, Rui Alves

**Affiliations:** 1 Nephrology Department, Centro Hospitalar e Universitário de Coimbra, 3000-548 Coimbra, Portugal; 2 Faculty of Medicine, University of Coimbra, 3004-531 Coimbra, Portugal; 3 Coimbra Histocompatibility Center, Portuguese Institute of Blood and Transplantation, 3041-861 Coimbra, Portugal; 4 Urology and Kidney Transplantation Unit, Centro Hospitalar e Universitário de Coimbra, 3000-548 Coimbra, Portugal

**Keywords:** chronic kidney disease, graft intolerance syndrome, immunosuppression, kidney graft failure, withdrawal

## Abstract

Patients with a failed kidney allograft have steadily increase in recent years and returning to dialysis after graft loss is one of the most difficult transitions for chronic kidney disease patients and their assistant physicians. The management of these patients is complex and encompasses the treatment of chronic kidney disease complications, dialysis restart and access planning, immunosuppression withdrawal, graft nephrectomy, and evaluation for a potential retransplant. In recent years, several groups have focused on the management of the patient with a failing renal graft and expert recommendations are arising. A review of Pubmed, ScienceDirect and the Cochrane Library was performed focusing on the specific care of these patients, from the management of low clearance complications to concerns with a subsequent kidney transplant. Conclusion: There is a growing interest in the failing renal graft and new approaches to improve these patients’ outcomes are being defined including specific multidisciplinary programs, individualized immunosuppression withdrawal schemes, and strategies to prevent HLA sensitization and increase retransplant rates.

## 1. Introduction

The last decade has seen a marked improve in short-term kidney transplantation outcomes, but long-term graft survival remains sub-optimal over the last 30 years [1]. This has led to an increased prevalence of kidney transplant (KT) recipients with graft loss, currently the fourth leading cause of incident dialysis, that will likely increase over time [2,3].

Returning to dialysis after graft loss is one of the most difficult transitions for chronic kidney disease (CKD) patients and their assistant nephrologists, as the conditions of these patients are complex and they face several complications. Patients with allograft loss have lower quality of life scores, a higher burden of depression, increased hospitalization, and significantly higher mortality rates [4,5]. From a health care provider point of view, the transition of care from transplant to dialysis is haphazard and not-standardized. The decision on the right timing to start dialysis, vascular access management, the optimal immunosuppression (IS) withdrawal, the impact of graft nephrectomy and relisting for a subsequent transplant are fundamental issues to address.

Despite the large number of patients affected by the deleterious consequences of graft loss, the only available recommendations on the care of the patient with a failing graft are the 2014 guidelines from the British Transplantation Society [6].

The management of the failing graft has become a growing concern in the kidney transplantation community and several groups are arising to address this complex issue. The development of multidisciplinary teams and a paradigm shift focusing on long term patient outcomes, combined with advances in immunosuppressive drugs and modern immunogenetic techniques are contributing to provide the best care to patients with graft loss. In the current review we aim to address the challenges in managing patient with a failing graft and review recent recommendations on this growing group of CKD patients.

## 2. Materials and Methods

This narrative review is based on an extensive literature research accessed between January and July 2022 from the following databases: NCBI Pubmed, ScienceDirect, the Cochrane Library and the websites clinicaltrails.org and uptodate.com. Full articles were accessed and reviewed. For the included articles, we used the tools “reference lists” and “related articles” of PubMed to increase our search. We used MeSH terms and free text, according to the specific chapter and in different combinations. The main keywords used were “allograft failure”, “chronic kidney disease”, “end stage renal disease”, “failing allograft”, “graft embolization”, “graft intolerance syndrome”, “graft nephrectomy”, “highly sensitized”, “immunosuppression”, “kidney transplant”, “retransplantation”, “sensitization”, “taper”, “withdrawal”. There were no restrictions on publication date but articles published in the last five years were prioritized. Only articles in English were selected.

## 3. Management of the Failing Allograft

### 3.1. Definition of a Failing Allograft

The definition of a failing allograft is the beginning of the challenge in managing KT recipients with decreased glomerular filtration rate (GFR). The Kidney Disease Improving Global Outcomes (KDIGO) clinical practice guidelines suggest that KT recipients with CKD stage 4T, in evidence of progression, should be prepared for kidney replacement therapy [7]. However, estimated GFR (eGFR) formulas have been derived from non-transplant patients and seem to be inaccurate in KT patients [8]. The GFR decline in patients with allograft dysfunction is also less predictable then transplant-naïve incident dialysis patients [9,10]. A recent study analyzed the variability of eGFR, assessed by most of the available equations, in reflecting measured GFR changes in KT patients, defined by repeated determinations of iohexol clearance. They found that eGFR, both by creatinine and/or cystatin-c formulas, was unreliable in reflecting real GFR changes over time [11].

A combined approach that includes eGFR trends, proteinuria, the presence of donor-specific antibodies (DSA), recurrent disease and transplant glomerulopathy might be the best predictor of graft failure [12,13]. A multicenter group have evaluated the four-variable (age, sex, eGFR and urine albumin-to-creatinine ratio) kidney failure risk equation in KT recipients, and concluded that the equation accurately predicts graft failure, especially in patients with eGFR < 45 mL/min/1.73 m^2^ [14]. The same results were found by a different group, which suggests that the use of this equation might help guide the nephrologist to make a decision on dialysis restart, living donation and the aggressiveness of anti-rejection therapies [15].

Another issue is the heterogeneity of causes of allograft failure and difficulty in defining the cause of graft loss. A recent study that thoroughly analyzed the cause of death-censored graft failure in 303 recipients, concluded that 51.2% of the patients had more than one cause contributing to graft loss and that the causes varied over time [16]. Focusing solely on the Banff criteria is difficult since most patients with progressive graft loss are not routinely submitted to late biopsies, and when biopsies are available, cumulative histological injuries coincide and accumulate. [17] Additionally, there are important contributors to graft failure such as aging or cardiovascular disturbances that are underrated in a histology focused practice [16,18].

**Figure 1:**
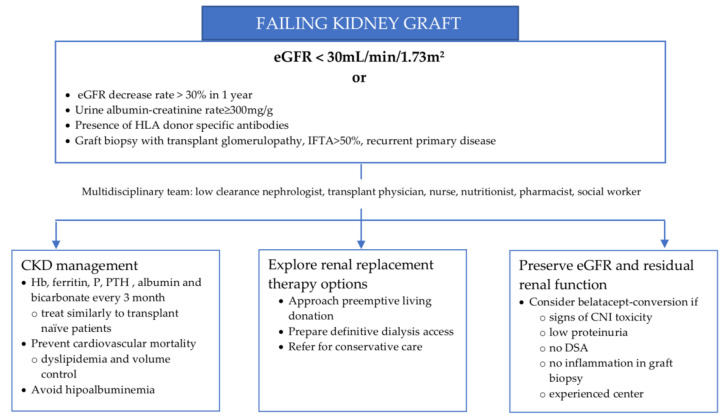
Managing the patient with a failing kidney graft. CKD—chronic kidney disease, CNI—calcineurin inhibitor, DSA—donor specific antibody, eGFR—estimated glomerular filtration rate, IFTA—interstitial fibrosis and tubular atrophy.

A group of experts proposed in 2021 that the failing allograft definition should include patients with stable but low allograft function; irreversible and progressive decline in kidney function with anticipated graft loss in less than 1 year; and a return to renal replacement therapy, maintenance dialysis, new wait-listing or repeat transplantation [19]. Figure 1 summarizes the management of the patient with a failing renal graft.

### 3.2. Chronic Kidney Disease Management

There are important dichotomies in CKD management between transplant-naïve patients and transplanted patients. The main focus of transplant nephrologists is to prolong allograft survival, which comes at the expense of a worst control of CKD-associated complications and a less organized beginning of dialysis. Higher blood pressure, anemia, CKD—mineral bone disorder (CKD-MBD) and worst nutrition contribute not only to higher morbimortality, but also play an important role in allograft dysfunction progression [20].

A large multicenter study from the UK Renal Registry investigated the prevalence of CKD-related complications in 1797 KT recipients with CKD stage 4 and 5. They concluded that renal allograft recipients had lower standards in anemia, CKD-MBD, blood pressure and nutrition than prevalent dialysis patients [21]. Another cross-sectional study, which included 72 renal transplant recipients with CKD-4 and 5T and compared them with eGFR matched transplant naïve patients, concluded that transplant patients were more likely to have uncontrolled and untreated hypertension, anemia and dyslipidemia [22]. There are several potential reasons why these patients perform worst, including resistance to therapy due to immunosuppression, chronic inflammatory state, and higher infection rates [21]. However, most authors agree that the main problem is the lack of standard of care, insufficient pre-dialysis education and inadequate communication between the transplant and dialysis teams.

Focusing on anemia, the benefit of using erythropoiesis-stimulating agents (ESA) and the optimum target hemoglobin remain uncertain in transplant patients [7]. A randomized clinical trial (RCT) evaluated the use of ESA in KT recipients with a mean eGFR of 35 mL/min/1.73 m^2^. The ESA group showed significantly higher levels of hemoglobin (12.5–13.5 g/dL vs. 10.5–11.5 g/dL) and a significantly slower rate of eGFR decline, without significant side effects [23]. A recent RCT that randomized 153 KT recipients to high or low hemoglobin targets (<12.5 vs. <10.5 g/dL) and to either cholecalciferol 1000 IU/day or control, showed that the 2-year decline in eGFR was lower in the high hemoglobin group but did not differ between cholecalciferol vs. control group [24].

The diagnosis and management of CKD-MBD in KT is very complex as it encompasses bone disease before KT, bone disease after KT, and the influence of immunosuppressant drugs, especially steroids [25]. KT recipients have a five-times-higher risk of bone fracture than general population, which represents an important contributor to morbidity and mortality [26]. There is no consensus on the most prevalent type of bone disease, and bone biopsy studies on KT recipients have small sample sizes and conflicting results [27,28,29]. The KDIGO guidelines recommend the evaluation of phosphorus and calcium every 1–3 months, and parathormone every 3–6 months in CKD-5 KT recipients and for patients with a higher risk of osteoporosis and bone mineral density assessment if results alter therapy. The recommended treatment for CKD-MBD is similar to patients without KT, but the level of evidence is “not graded” [30]. We believe that an individualized approach to CKD-MBD in patients with a failing graft is the key to success, and must consider the treatment of hyperparathyroidism, the prevention of osteoporosis, and the potential harm of prolonging steroids versus the acute rejection of failed graft.

Volume overload and dyslipidemia may also be important contributors to the progression of graft dysfunction and cardiovascular mortality. Data from the UK renal registry showed that the majority of KT recipients with eGFR < 30 mL/min/1.73 m^2^ did not reach target levels regarding blood pressure or LDL levels [21].

A definitive dialysis access, especially for hemodialysis (HD), is another important issue in KT recipients returning to dialysis. Several authors have reported that KT recipients start HD by a CVC more frequently than arteriovenous fistula, and a large registry study concluded that 65% of patients restart dialysis using a CVC [31,32]. Considering that KT patients are immunosuppressed and have a higher incidence of infection, these findings are alarming and need to be addressed [33]. A recent large cohort study concluded that resuming dialysis with a CVC is an independent risk factor for mortality after graft loss [4]. The worst management of CKD-related disease continues more than 1 year after dialysis resuming, and patients with a previous KT have the worst dialysis quality metrics compared to transplant-naïve patients, having a major impact on morbidity and mortality [4,34,35].

### 3.3. Individualized Care to a Patient with a Failing Allograft and Strategies to Defer Dialysis

The British Transplantation Society recommends the development of dedicated low clearance transplant Clinics, which provide multidisciplinary tailored care from both a transplant and a low clearance perspective, including specialized nephrologists, nurses, dietitians and pharmacists [6]. Two studies accessed the benefits of low clearance transplant clinics in graft failure recipients’ outcomes. No differences were found regarding clinical or biochemical parameters, retransplantation or mortality, but patients followed in low clearance clinics received more counselling regarding dialysis modality, less unplanned dialysis starts and more prompt transplantation work-up [36,37].

This individualized multidisciplinary approach is more focused on long-term outcomes and brought some novelties with regard to differing eGFR decline, prolonging residual renal function and improving patients’ quality of life [38].

Focusing on stabilizing eGFR, calcineurin inhibitor (CNI)-free regimens could be beneficial considering their nephrotoxicity and metabolic side effects, despite a higher risk of acute rejection and HLA sensitization [39,40,41,42]. The BENEFIT and BENEFIT-EXT trials have shown that de novo belatacept-based immunosuppression is associated with better renal function and less chronic allograft nephropathy compared with cyclosporin schemes [43,44]. The benefits of belatacept conversion in patients with longer post-transplant time to attenuate previous CNI toxicity is not well-established. A retrospective study analyzed the role of belatacept as a rescue therapy in patients with chronic graft dysfunction (mean eGFR = 22 ± 9.4 mL/min/1.73m^2^) and found a significant increase in eGFR (32 ± 13 mL/min/1.73m^2^), improvement in serum bicarbonate levels, better CKD-MBD control, and an increase in albumin levels [45]. In a larger cohort, conversion to belatacept in patients with eGFR < 30 mL/min/1.73m^2^, was only beneficial in a subgroup of patients with less time post-transplant, low proteinuria, and less HLA sensitization [46]. Another group explored the conversion to belatacept in patients with a mean post-transplant time of 10.6 ± 7.6 years. They found similar graft loss rates but better eGFR at two year follow-up, with the exception of patients with severe eGFR impairment and Banff microvascular inflammation [47]. These results suggest a potential benefit in late conversion to belatacept, especially in patients without chronic allograft nephropathy. Belatacept is intravenously administered monthly, which may represent a practical benefit to the pluri-medicated patient with a failing graft and address drug adherence issues.

Incremental HD strategy is another novelty with the goal of preserving residual renal function on patients with a failing graft. Although there are no published RCT studies available, recent recommendations believe that it might be beneficial by extrapolation of native kidney CKD patients [38,48].

### 3.4. Dialysis Timing, Modality and Conservative Care

Studies on the optimal timing of dialysis initiation after graft failure are lacking. A cohort study that evaluated 4741 patients with graft failure showed that higher eGFR at dialysis restart was associated with higher mortality [49]. However, there was an important confounder since the most ill patients with more comorbidities started dialysis sooner. Molnar et al. used a propensity score analysis to evaluate the outcomes of 747 patients returning to dialysis. The fully adjusted model showed that eGFR was not associated with the risk of death but found a trend towards higher mortality risk with earlier dialysis initiation in the youngest, healthiest patients [50]. These findings are in agreement with the results of the IDEAL study in native kidney CKD [51]. More studies are needed to address the best timing for starting dialysis since isolated eGFR is not a good marker.

The majority of patients with graft loss who resume dialysis opt for HD, but when compared to native kidney patients, there are more KT recipients choosing peritoneal dialysis (PD), probably due to age and autonomy bias [52,53]. A large, matched cohort study, performed by the French Language Peritoneal Dialysis Registry, compared 328 PD incident patients who experienced graft loss with 656 matched transplant-naïve patients and concluded that, despite similar peritonitis rates, transplant patients had a significantly higher rate of PD technique failure. The main reason for HD transfer was adequacy and/or ultrafiltration failure [54]. Another smaller study, found a small but significantly higher rate of PD technique failure in the failed-graft group, which was not related to peritonitis or peritoneal membrane failure [55], and a recent small retrospective study showed no differences in survival technique, peritonitis-free survival or residual renal function between graft failure and transplant-naïve patients. Another interesting finding was that patients with graft failure that maintained low dose IS had more preserved residual renal function across the first year of PD [56]. Several authors compared HD with DP in patients returning to dialysis after a KT, and most studies show similar early and overall survival [53].

Kidney palliative care is an emerging subspecialty of palliative care and nephrology, but for KT recipients, the utility of kidney palliative care has not been explored nor well-delineated. A recent small trial published by a group of inpatient kidney palliative care service (KidneyPal) showed that providing a trained and multidisciplinary team of palliative care to patients with graft failure increased the adherence of patients to palliative care and time-limited trial dialysis, resulting in a more active role of the patient to decide their future after graft loss [57].

### 3.5. Immunosuppression Withdrawal

Decisions on tapering immunosuppressive medications in patients returning to dialysis are complex, without a strong evidence base or universally accepted recommendation, resulting in widely clinical practice variance. The Kidney Recipient with Allograft Failure Transition of Care (KRAFT) group distributed an online questionnaire to 92 KT centers to evaluate the patterns of management of patients with renal allograft, concluding that practices in the US vary greatly [58]. Several studies have addressed the impact of IS withdrawal after KT failure, but the majority are small and retrospective or registry analyses with limited information on IS withdrawal. The first large observation study analyzed 119 consecutive patients with allograft failure and PRA < 20%, concluding that weaning IS was a triggering event to graft intolerance syndrome (GIS) and an independent predictor of HLA sensitization independent of nephrectomy [59]. Other small retrospective studies have conflicting results, either in favor of [60,61] or opposed to IS maintenance [62,63]. More recently, a retrospective single-center study that included 131 KT patients starting dialysis post-graft failure concluded that IS maintenance with more than two drugs was an independent risk factor for mortality and found no differences in GIS nor retransplantation. A significant limitation of this study was a big heterogeneity regarding IS maintenance protocol, including 8 patients (36%) that maintained triple therapy for more than 1 year [64]. Another recent cohort showed that maintaining IS with two drugs significantly decreased the risk of developing HLA antibodies, determined by Luminex single-bead assays, and that the majority of the patients developed HLA-specific antibodies >12 months after relisting, which reflected IS withdrawal [65]. Another retrospective study published in 2021, which included 134 patients, compared IS weaning in three timings: <90 days, 90–180 days and >180 days, concluding that prolonged IS withdrawal did not reduce sensitization or improved retransplantation rates but decreased the risk of graft nephrectomy [66].

The first prospective multicenter study on IS withdrawal was recently published and included 269 patients with graft loss from 16 Canadian centers. Two groups were compared at a two-year follow up period: group 1—continuation of IS with CNI and/or antiproliferative and/or prednisone versus group 2: discontinuation of all IS or use of prednisone only. In multivariable models, group 1 patients had a lower risk of death and no differences in hospitalization due to infection. Group 1 presented less HLA antibodies determined by Luminex, especially class 2, but did not reach significance. There were no differences in virtual panel reactive antibody (PRA) or GIS [67].

Despite the conflicting results presented by the available studies, groups of experts are proposing strategies to IS withdrawal in patients with a graft loss (Table 1).

These recommendations mainly focus on three aspects: the possibility of a retransplantation; the adverse effects of IS, including cardiovascular disease, infection and neoplasia; and the preservation of residual renal function.

A major difficulty in maintaining IS after graft loss is the adequate dosing of CNI. In a study by Augustine and colleagues, the majority of the patients who continued IS had a functioning pancreas graft and maintained higher levels of CNI, which might explain the lower HLA sensitization [59]. Additionally, a recent trial that included 45 patients with graft loss that maintained tacrolimus for 24 months, showed that higher tacrolimus levels (≥3 mg/dL) were protective against allosensitization [61]. Lubetzky et al. determined tacrolimus as 3–5 ng/mL [19].

Innovative approaches to IS withdrawal after graft loss in order to decrease sensitization are arising. A pilot RCT published in 2021, randomized 13 patients with failed allograft upon reinitation of dialysis to belatacept versus IS discontinuation and evaluated HLA antibody formation 36 months after randomization. Overall, the breadth and depth of HLA antibody formation was greater in the withdrawal group relative to belatacept treated patients, with similar adverse events [73]. These preliminary findings suggest a role of belatacept on preventing allosensitization after graft loss.

### 3.6. Graft Nephrectomy

There are formal indications for graft nephrectomy that the majority of the centers follow (Table 2), but for the remaining patients there is no consensus on timing and indications for allograft nephrectomy.

When an established indication of graft nephrectomy is not present, the reported rate of surgical allograft nephrectomy after graft failure varies from 9 to 74%, depending on the center policy rather than compelling data [74,75]. The potential benefits of allograft nephrectomy include the prevention of GIS, the possibility of IS withdrawal, and making more room for a new graft. The risks are loss of residual renal function, HLA sensitization, and surgical morbidity and mortality [76]. There are two opposing theories concerning HLA sensitization and graft nephrectomy: the failing graft can trigger HLA antibodies formation due to the continued exposure of non-self-antigens versus the “sponge theory” that the graft absorbs anti-HLA antibodies and decreases their serum detection [75,77]. In most cohorts, peak PRA levels are higher in patients who undergo allograft nephrectomy, including studies using single-antigen assays [63,65,75,78]. However, in many cases, graft nephrectomy occurs in the context of GIS after IS withdrawal, which might be a major sensitizing event [59].

A recent meta-analysis explored the impact of allograft nephrectomy in 2256 patients with subsequent kidney retransplantation, concluding that a prior allograft nephrectomy increased the risk of higher PRA, delayed graft function, acute rejection and primary non-function but had no significant association with five-year graft or patient survival [79]. While one study showed that allograft nephrectomy improved mortality, suggesting that the avoidance of the chronic inflammatory state and side effects of IS are beneficial, the majority of the studies found no differences in mortality between patients with or without graft nephrectomy [80]. A possible benefit of early and systematic transplantectomy versus a gradual reduction in immunosuppression is currently under investigation in a multicentric RCT [81].

Another interesting approach that bypasses the surgical risk of allograft nephrectomy is the embolization of the renal artery. A recent meta-analysis showed that renal artery embolization successfully treated GIS in the majority of the patients, with lower morbidity and mortality, but 20% of the patients required nephrectomy at follow-up [82]. Post-embolization syndrome, described as low-grade fever, local pain, malaise and increased inflammatory parameters frequently occurs, but is generally mild and can be treated with short courses of steroids [83]. Regarding HLA sensitization, the rates of transfusion are lower with embolization, but the impact on alloimmunization is currently unknown [78]. If no stablished indications for graft nephrectomy are present, the option of percutaneous embolization should be considered, especially in patients with a high surgical risk and PD patients, in order to decrease the risk of peritoneal membrane injury.

### 3.7. Relisting for a Subsequent Kidney Transplant

In selected patients with appropriate clinical conditions to receive a new graft, retransplantation offers the largest survival benefit, with a mortality rate reduction ranging from 20% to 88%, depending on specific comorbidities and transplant era [84,85,86]. The British Transplant Society Guidelines recommend that patients suitable for retransplant should be evaluated when graft failure is anticipated within the next year, and ideally provide a preemptive retransplantation [6]. However, the access to a subsequent KT is frequently compromised by HLA sensitization, and a large number of young CKD patients with a previous KT are constantly waiting for a subsequent graft [87]. The access to retransplantation is a very complex and intricate topic that involves not only the assistant nephrologist and transplant surgeon, but also the immunologist and regional allocation laws. This topic is discussed in detail in another review article [88].

## 4. Conclusions

With the increasing number of KT recipients returning to dialysis, the nephrology community is shifting towards a better paradigm and most transplant centers are now driven to deliver long-term, individualized care. The patient with a failing graft encompasses several issues that are similar to native kidney CKD patients, but longer dialysis vintage and exposure to IS make this population management more complex. New data on IS withdrawal management, impressive progresses in immunogenetics and new pharmacological therapies are arising and bringing a new hope to CKD-5 patients with a failing graft.

## Figures and Tables

**Table 1:** Expert recommendations for immunosuppression withdrawal after kidney graft failure.

	Candidate for Retransplant	Not a Candidate for Retransplant	Other Notes
Lubetzky et al. (KRAFT) AST 2022 [19]	Reduce anti-metabolite by 50% and maintain CNI ± low-dose PDN **3 months:** stop anti-metabolite, low-dose CNI ± low-dose PDN **6 months:** reduce CNI by 50% ± low-dose PDN **9 months:** reduction in CNI or maintenance of PDN 5 mg **12 months:** stop of all IS	Stop anti-metabolite and taper CNI by 50% and/or low-dose PDN therapy for 6–12 months	Monitor patient every 3–6 months until patient is off IS Monitor sensitization while wait-listed
Davis et al. CJASN 2022 [68]	Continue CNI, stop anti-metabolite, and reduce PDN by 1 mg/month until discontinued **3–6 months:** reduce CNI by 50% and reduce PDN **6–12 months:** stop CNI and reduce PDN **If no residual function is present:** stop CNI, continue anti-metabolite or reduce by 50%, and reduce PDN by 1 mg/month until discontinued **1–2 months:** Reduce anti-metabolite by 50% or discontinue and reduce PDN **2–3 months:** stop anti-metabolite and reduce PDN	Stop CNI and anti-metabolite and reduce PDN by 1 mg/month until discontinued	If repeat transplant is expected within 1 year, continue IS with a 50% reduction in anti-metabolite
Miller; Brennan UptoDate [69]	**If retransplantation planned within 1 year:** stop antimetabolite, reduce CNI dose to once daily and continue until retransplant, maintain PDN 5 mg/day. **If retransplantation not likely within 1 year:** **urinary output > 400 mL:** stop antimetabolite, taper CNI slowly over 6–12 months, taper PDN by 1 mg/moth until discontinued **urinary output < 400 mL:** stop antimetabolite, taper CNI over 3–6 months, taper PDN by 1 mg/ month until discontinued	Similar to recommendation for patients unlikely to undergo retransplantation within 1 year	If early graft failure (<1 year), preemptive transplant nephrectomy and complete withdrawal of IS Tacrolimus 24 h trough levels 2–5 mg/mL and cyclosporine 50–75 ng/mL.
Fiorentino et al. CKJ 2021 [70]	If residual renal function is not preserved, taper IS until discontinued If residual renal function is preserved, continue low-dose IS Maintain steroids 5 mg/daily	The same as recommendation for retransplant candidates	If preemptive retransplantation is available, continue IS therapy Withdrawal recommendation similar to that of British Transplantation Society
Kassakian et al. NDT 2016 [71]	**If retransplant < 1 year:** stop CNI or mTOR immediately, wean antimetabolite over 3 months and maintain pPDN 5 mg daily	Stop antimetabolite and CNI/mTOR and wean PDN 1 mg/day for 1 month If residual renal function is preserved, 0.5 mL/min (the same as candidates for retransplant)	If infection requiring hospitalization or graft nephrectomy, stop IS Check PRA immediately upon return to dialysis and monthly if plans to retransplant.
Pham et al. World J Nephrol 2015 [72]	**If antimetabolite + CNI + PDN:** Discontinue antimetabolite, taper CNI over 4–6 weeks, maintain same steroid dose until 2–4 weeks, and then taper 1 mg/month until discontinued **If CNI + mTOR + PDN:** Discontinue amTOR, taper CNI over 4–6 weeks, maintain same steroid dose until 2–4 weeks, and then taper 1 mg/month until discontinued **If mTOR + PDN:** Taper mTOR over 4–6 weeks, maintain same steroid dose until 2–4 weeks, and then taper 1 mg/month until discontinued	If urinary output > 0.5–1 L/daily and low-risk patient without significant comorbid condition, the same as recommendation for retransplant candidate	If significant comorbidities, stop IS If living donor available, maintain all IS
British Transplantation Society 2014 [6]	Stop antimetabolite immediately, gradual taper of CNI or mTOR with a 25% dose reduction per week, steroids should be the last to be withdrawn, 1 mg/month once the dose is below 5 mg/day	The same as recommendation as for retransplant candidate	If repeat transplant expected within 1 year, continue IS If transplant nephrectomy, stop all IS immediately (taper steroids)

CNI—calcineurin inhibitor; PDN—prednisone; mTOR—mTOR inhibitor.

**Table 2:** Indications for graft nephrectomy after graft loss [6,66,67,70].

Established Indications for Graft Nephrectomy
Primary dysfunction
Severe acute rejection resistant to immunosuppression
Severe graft pyelonephritis/urosepsis
Post-transplant lymphoproliferative disease in the graft
Refractory graft intolerance syndrome
Graft hemorrhage or thrombosis
To create space for subsequent retransplant
**Relative Indications for Graft Nephrectomy**
Early graft loss (<6–12 months)
Resistant BK virus nephropathy

## Abbreviations

CKD	chronic kidney disease
CKD-MBD	chronic kidney disease associated mineral bone disorder
CNI	calcineurin inhibitor
cPRA	calculated panel reactive antibody
DSA	donor specific antibody
ESA	erythropoiesis stimulating agents
GFR	glomerular filtration rate
GIS	graft intolerance syndrome
HD	hemodialysis
IS	immunosuppression
KDIGO	kidney disease improving global outcomes
KT	kidney transplant
KRAFT	the kidney recipient with allograft failure transition of care
PD	peritoneal dialysis
PRA	panel reactive antibody
RCT	randomized clinical trial
